# Pericapsular Hip Block Guided by Ultrasonography in Elderly People with Hip Fracture in the Emergency Sector: Clinical Trial

**DOI:** 10.1055/s-0044-1785494

**Published:** 2024-04-10

**Authors:** Gustavo Martins Fontes, Marcelo Vaz Perez, Edson Hidenori Miashiro, Antonio Isidoro de Sousa Neto, Thiago Ramos Grigio, Luiz Henrique Silveira Rodrigues

**Affiliations:** 1 Departamento de Ortopedia e Traumatologia, Hospital Municipal Dr. Alípio Corrêa Netto (HMACN), São Paulo, SP, Brasil; 2 Serviço de Terapia da Dor, Faculdade de Ciências Médicas da Santa Casa de São Paulo, São Paulo, SP, Brasil; 3 Faculdade de Ciências Médicas da Santa Casa de São Paulo, São Paulo, SP, Brasil; 4 Disciplina de Ortopedia e Traumatologia, Faculdade de Medicina, Fundação Educacional do Município de Assis (FEMA), Assis, SP, Brasil; 5 Grupo de Dor, Instituto do Câncer do Estado de São Paulo (ICESP), São Paulo, SP, Brasil; 6 Faculdade de Medicina de Jundiaí, Jundiaí, SP, Brasil

**Keywords:** analgesia, anesthesia, conduction, nerve block, pain, hip fractures, ultrasonography

## Abstract

**Objectives** This study evaluated pain intensity in elderly subjects with hip fractures admitted to the emergency sector and undergoing preoperative pericapsular nerve group (PENG) block. Additionally, the degree of tolerable hip flexion was assessed.

**Methods** A prospective, randomized, and controlled clinical trial with parallel groups. The control group consisted of elderly subjects with hip fractures undergoing standardized intravenous systemic analgesia. The intervention group consisted of elderly patients with hip fractures undergoing PENG block and standardized systemic analgesia. The groups were evaluated at rest and during movement using the Pain Assessment in Advanced Dementia (PAINAD) scale. We determined pain intensity and reduction, in addition to the degree of tolerable flexion of the fractured hip. All patient assessments occurred before the medication or block administration and at 45 minutes, 12, 24, and 36 hours postmedication or block.

**Results** Preoperatively and 24 hours after PENG block, elderly subjects with hip fracture showed a significant reduction in pain at rest or movement compared to control patients ( *p* < 0.05), with 60% of patients assessed at rest demonstrating desirable pain reduction (≥50%) and only 13.3% of the control group achieving the desired pain reduction. During movement, after undergoing PENG block, 40% of subjects demonstrated the desired pain reduction and no patient from the control group. The intervention group also showed a significant improvement in the tolerable hip flexion group ( *p* < 0.05).

**Conclusion** Preoperative PENG block in elderly subjects with hip fractures admitted to the emergency sector provided a significant reduction in pain compared with the control group.

## Introduction

Proximal femoral fractures are common in the elderly population. 1 Their prevalence is increasing given the growing longevity of the population, 2 with an estimated global incidence of 6.3 million elderly subjects by the year 2050. 3

These fractures are an orthopedic emergency with significant mortality and morbidity and require surgical treatment and adequate analgesia. 3 4 Surgery is recommended, preferably within the first 24 to 48 hours, as it demonstrates pain relief and reduces the incidence of postoperative complications and mortality. 5 6

Unfortunately, in public emergency services, it is not uncommon to find elderly patients with hip fractures in bed waiting for definitive surgical treatment and experiencing severe pain. In this preoperative period, the administration of opioids for pain relief is the usual therapy, even though it is associated with several side effects, such as nausea, vomiting, constipation, hypotension, drowsiness, and mental changes. Less commonly, but even more worrying, is that some patients can develop delirium and life-threatening respiratory depression. 4 7 In contrast, the fear of these complications by the medical and nursing team can increase the risk of oligoanalgesia. The use of opioids in elderly patients requires a balance based on their harmful potential and inefficiency when administered parenterally alone. 8 Inadequate pain control or excessive opioid use are directly linked to an acute confusional state, 8 which, in turn, when associated with hip fractures, can virtually double the mortality rate in one year in this population. 9

Therefore, pain treatment, in addition to being a humanitarian issue, impacts the good outcomes of these patients. Pain is associated with increased neurohormonal stress response, myocardial ischemia, and delayed recovery and mobilization in these patients. 7 The literature review emphasizes that analgesia in the elderly population should focus on minimizing risk factors for delirium, including pain, constipation, and delirium-like side effects. 10

Within this context, regional anesthesia for the management of acute pain is increasingly present in emergency sectors or departments, 8 demonstrating better efficacy compared with the traditional analgesia available to patients with hip fractures. 4 Hip regional blocks performed in the emergency sector demonstrated benefits in reducing pain and opioid use, being recommended by a systematic review. 11 However, the association with ultrasound (US) contributes to the efficiency of this technique. 4 12

**Fig. 1:**
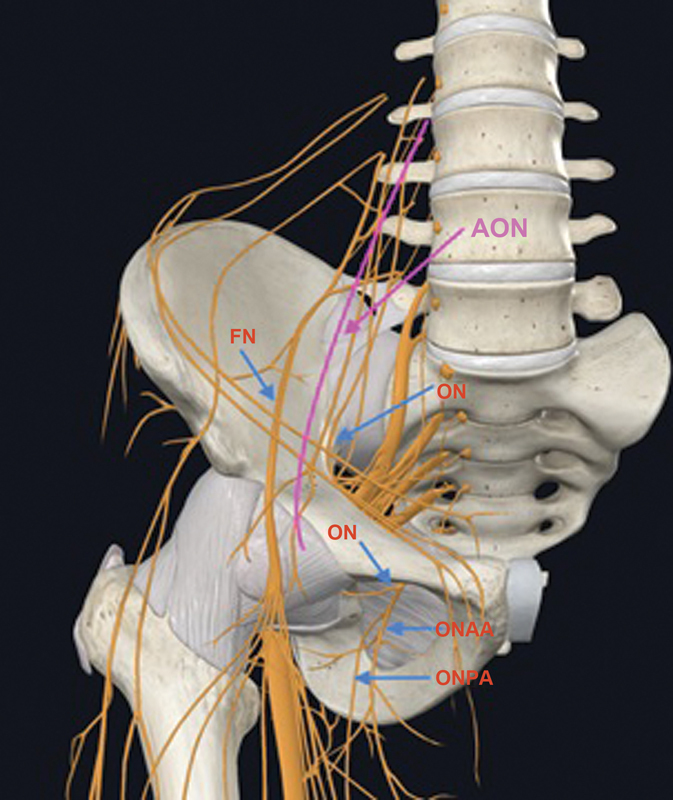
Innervation of the anterior region of the hip joint capsule. **Abbreviations:** FN, Femoral nerve; ON, obturator nerve; AON, accessory obturator nerve; ONAA, obturator nerve, anterior branch; ONPA, obturator nerve, posterior branch. 15

**Fig. 2:**
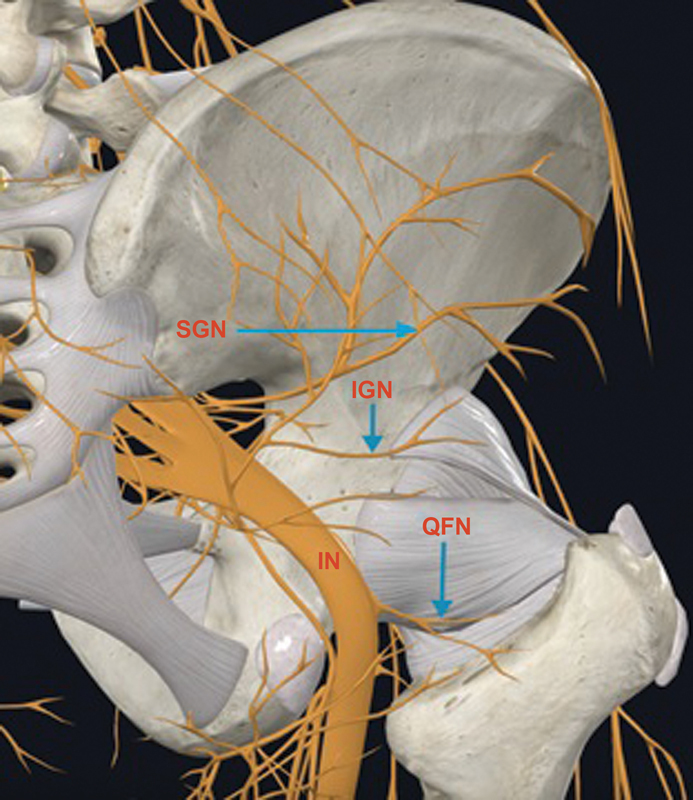
Innervation of the posterior region of the hip joint capsule. **Abbreviations:** IGN, Inferior gluteal nerve; SGN, superior gluteal nerve; IN, ischial nerve; QFN, quadratus femoral nerve. 15

Understanding the anatomical aspect of the joint capsule is critical for effective hip analgesia. The anterior region of the capsule receives innervation from the femoral nerve (FN), obturator nerve (ON), and accessory obturator nerve (AON) branches, which are the major contributors to the sensory innervation of the hip joint. This innervation pattern suggests that these branches must be the main block targets. 13 14 15 ( Fig. 1 and 2 )

**Fig. 3:**
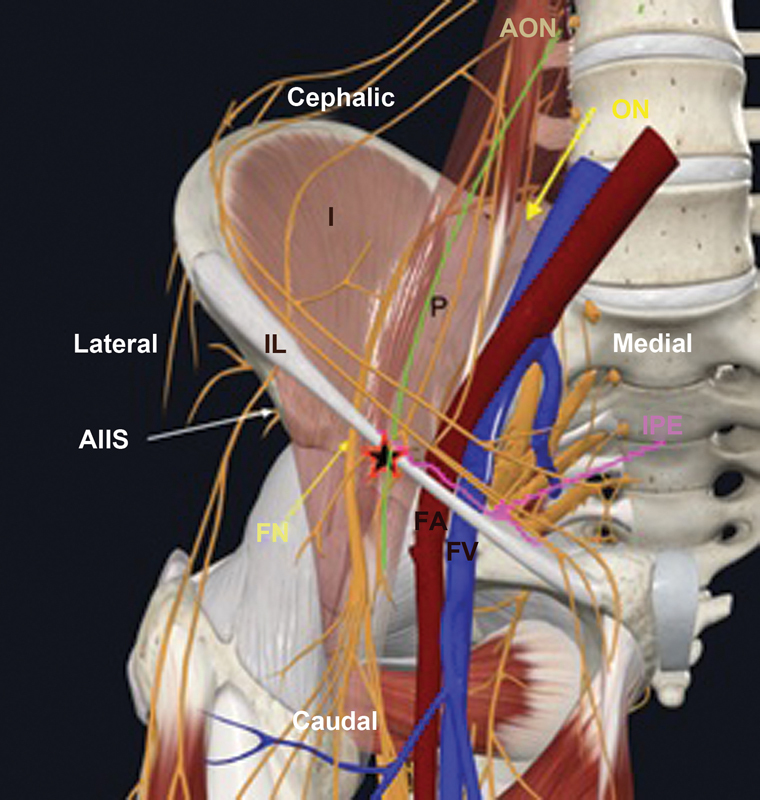
Anatomical location of the PENG block in a three-dimensional representation of the right hemipelvis and hip. **Abbreviations:** FA, Femoral artery; AIIS, anterior inferior iliac spine; IPE, iliopubic eminence; I, iliacus muscle; IL, inguinal ligament; FN, femoral nerve; ON, obturator nerve; AON, accessory obturator nerve; P, psoas major muscle; FV, femoral vein;  anesthetic infusion point; PENG, pericapsular nerve group. 15

**Fig. 4:**
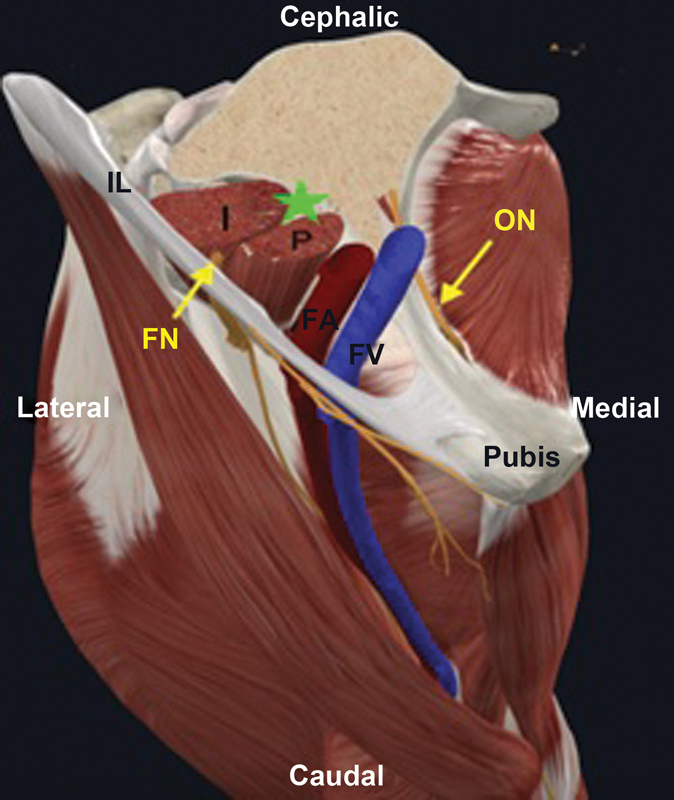
Anatomical location of the PENG block in a three-dimensional representation of the right hip (transverse section). **Abbreviations:** FA, Femoral artery; I, iliacus muscle; IL, inguinal ligament; FN, femoral nerve; ON, obturator nerve; P, psoas major muscle; FV, femoral vein;  anesthetic infusion point; PENG, pericapsular nerve group. 15

Girón-Arango et al., 14 based on this anatomical information and considering that the main FN and AON branches consistently lie between the anterior inferior iliac spine and the iliopubic eminence, described an US-guided technique for blocking these capsular branches of the hip, known as pericapsular nerve group (PENG) block. 14 15 16 ( Fig. 3 and 4 )

Preoperative PENG block reports in elderly subjects with hip fractures, performed in the public service emergency sector, are scarce. In this scenario, the present study considers that this block could help reduce pain and increase the mobility of these patients compared with standardized systemic analgesia.

The primary objective of this study was to evaluate pain intensity in elderly patients with hip fractures admitted to the emergency sector and undergoing preoperative PENG block. Our secondary goal was to assess the tolerable degree of flexion of the affected hip.

## Material and Methods

This prospective, randomized, controlled clinical trial with two parallel arms was registered on Plataforma Brasil and approved by the Research Ethics Committee (protocol number 38115120.4.0000.5479). All patients or legal guardians signed an Informed Consent Form. The study was registered under number RBR-2zdn8pb in the Brazilian Clinical Trials Registry (REBEC).

Patient selection among elderly subjects admitted to the emergency sector of a public hospital with a radiographic diagnosis of hip fracture was sequential, using stratified probabilistic sampling. Type I error was set at 0.05, with *p1* = 0.05 (proportion of subjects from the control group with at least 50% reduction in pain during movement) and *p2* = 0.5 (proportion of subjects in the intervention group with at least 50% reduction in pain during movement), and the test power was 0.80. The total sample consisted of 30 patients. 17 However, considering a 5% dropout rate, the optimal final sample for the study was of 32 patients ( Table 1 ).

**Table 1:** Sample size for *p1* = 0.05 and *p2* = 0.50

Type I (α) error	Statistical power (1-β)	* p _1_*_ _ = 0,05 and * p _2_*_ _ = 0,50			
		n	n per group	+5%	
				n	n per group
	0.80	30	15	32	16

We approached 59 patients with hip fractures for potential inclusion in the study from March 2020 to February 2022.

### Inclusion Criteria

Patients aged 65 years or older, regardless of gender or level of cognition, radiographically diagnosed with acute type III or IV femoral neck fracture based on the Garden classification, 18 or with type III to V transtrochanteric fracture per the Tronzo classification, 19 and with American Society of Anesthesiology (ASA) classification II and III, were included. 20

### Exclusion Criteria

Patients with chronic, pathological fractures, other fractures, previous hip flexion limitation, history of allergies or reported reactions to the anesthetic used for the block, or skin lesions close to the puncture site were excluded. We also excluded patients with advanced renal or hepatic failure, those using anticoagulants (therapeutic dose) or presenting a coagulation disorder before the fracture, as well as patients unaccompanied by their legal guardians.

There were 27 patients excluded, and 32 were randomized into four blocks using a computer-generated random numerical sequence 21 placed in a sealed envelope. After opening the envelope, a researcher not linked to the study performed the draw at the time of hospitalization. Since this study was open, patients and investigators knew the allocation group after the draw. Only the principal investigator performed the PENG block, while previously trained investigators and residents collected the data.

**Fig. 5:**
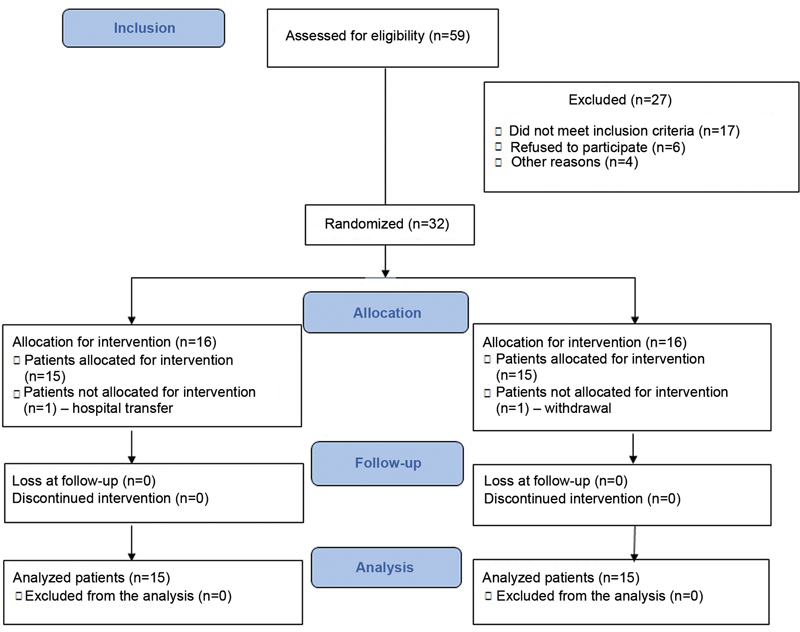
A CONSORT 2010 flowchart.

The control and intervention groups had 16 patients each. At the end, 15 subjects from each group were assessed, totaling 30 patients ( Fig. 5 ).

### Analyzed Variables

- Sociodemographic data and fracture types.
- Pain Assessment in Advanced Dementia (PAINAD) scale's score at rest and movement.
- Tolerable degree of flexion of the fractured hip.

**Fig. 6:**
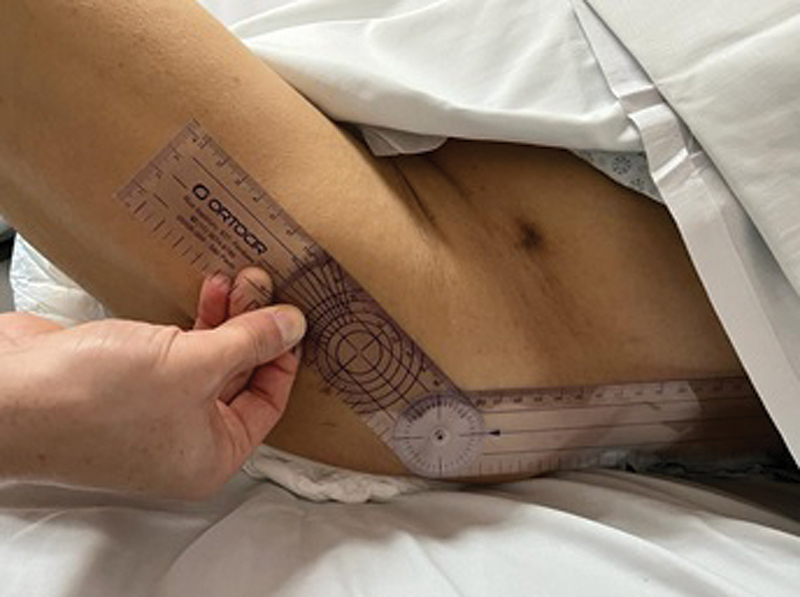
Measurement of tolerable hip flexion using a goniometer.

We applied the same form to all patients, which included the following information:

a) Sociodemographic and clinical data: including age, gender, vital signs, preexisting diseases, ongoing medications, previous treatment, personal history, habits and addictions, drug allergy, fracture type, signs of systemic toxicity, adverse effects, or complications.
b) Pain assessment per the translated and validated PAINAD, 22 an observational scale consisting of five items: breathing, negative vocalization, social expressions, body language, and comfortability. The score for each item ranges from 0 to 2, with a total score from 0 to 10 points. 23
c) Tolerable hip flexion according to a goniometer measurement on the fractured hip during passive and assisted flexion with neutral rotation and interrupted by signs of pain, resistance to movement, or whichever occurs first. The measurements were stratified as 0 to 15, 16 to 45, 46 to 60, and > 60° flexion ( Fig. 6 ).

### Control Group

We assessed subjects at rest and during movement before administering any medication using the research questionnaire and recorded pain intensity and tolerable hip flexion.

Subsequently, these patients received systemic intravenous analgesia (tramadol, 100 mg, and sodium dipyrone, 1 g, every 8 and 6 hours, respectively). Reassessment occurred at rest and movement after 45 minutes, 12, 24, and 36 hours.

### Intervention Group

**Fig. 7:**
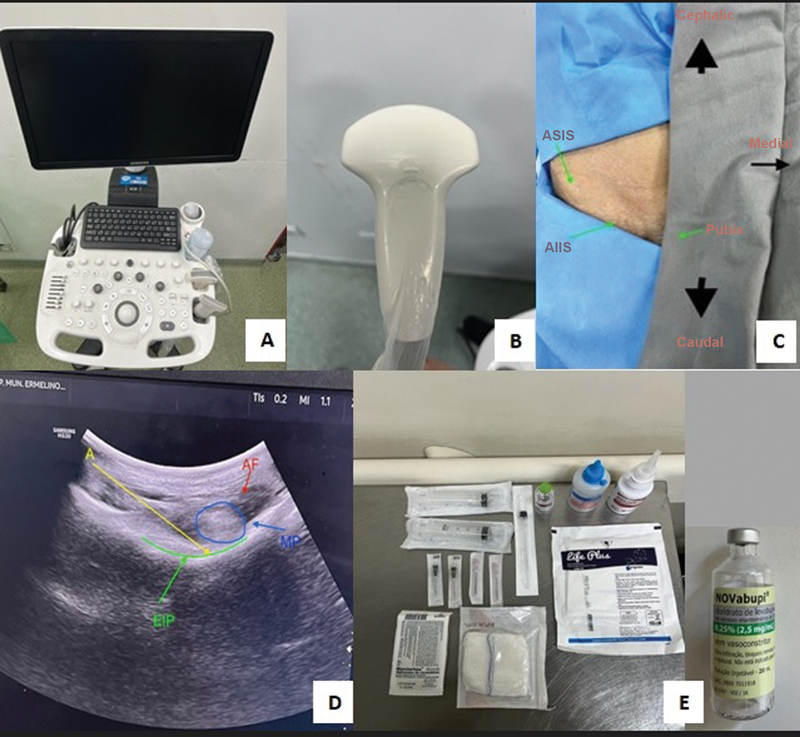
Ultrasound and patient preparation for the PENG block. ( **A** ). Ultrasound device used in the study. ( **B** ). Curvilinear transducer used in the study. ( **C** ). Hemipelvis with sterile fields to receive the PENG block. ( **D** ). Ultrasound image during PENG block. ( **E** ). Material used for the PENG block. **Abbreviations:** A, Site of local anesthetic application; FA, femoral artery; AIIS, anterior inferior iliac spine; ASIS, anterior superior iliac spine; IPE, iliopubic eminence; P, psoas major muscle; PENG, pericapsular nerve group.

The intervention group was assessed before the PENG block following the same protocol used for the controls. Next, these patients went to the emergency sector's procedure room to perform the US-guided PENG block. After asepsis and antisepsis of the fractured hip, the investigator, wearing a mask and sterile gloves, placed a sterile field and sterile protection for the ultrasound low-frequency curvilinear transducer model HS30, identification code US591, (Samsung Ltd., Suwon, South Korea) under regular maintenance. With the patient in the supine position, ultrasound visualization identified the anterior superior iliac spine (ASIS), anterior inferior iliac spine (AIIS), iliopectineal eminence (IPE), femoral artery and vein, and psoas tendon. After confirming the anatomical references, the investigator performed an anesthetic button by introducing a 100 mm, 22G needle from lateral to medial, immediately lateral, and inferior to the psoas, until the IPE was reached. After negative aspiration to rule out intravascular introduction, 25 mL of a 0.25% levobupivacaine anesthetic solution were administered ( Fig. 7 ).

After the PENG block, at 45 minutes, 12, 24, and 36 hours, the research questionnaire was administered again at rest and during movement, recording pain intensity and tolerable degree of hip flexion for each period. This group also received the same systemic analgesia as the control.

### Primary and Secondary Outcome

Outcomes included a reduction in pain intensity by ≥ 50% using the PAINAD scale in the preoperative period of elderly subjects with hip fractures undergoing the PENG block and improvement in tolerable hip flexion ≥ 45° per goniometer assessment.

### Statistical Analysis

Patient characteristics were presented as absolute and relative frequencies (for qualitative variables), and mean, median, standard deviation (SD), minimum, maximum, as well as first- and third-quartile values.

The Mann-Whitney test compared quantitative variables from independent groups. The Pearson chi-square or Fisher exact test assessed the association between qualitative variables.

The McNemar test evaluated the degree of flexion frequencies before and after the intervention.

We adopted a 5% significance level for all hypothesis tests and performed the analyses using the statistical software Statistical Package Social Sciences (SPSS, IBM Corp. Armonk, NY, USA) for Windows, v.25. Result presentation followed the study objectives:

- Comparison of the PAINAD between groups and times.
- Comparison of PAINAD variation between times per group.
- Comparison of pain reduction by ≥ 50% between groups and time.
- Assessment of the degree of flexion between groups and times.
- Comparison of PAINAD variation and fracture types.
- Description of the sociodemographic and clinical characteristics of the study participants according to the assigned treatment.
- Graphical representation of results.

## Results

There were no statistically significant differences regarding sociodemographic and clinical characteristics ( Table 2 ).

**Table 2:** Characteristics of study patients per treatment group

Characteristic	Control	Intervention	Total	*p* -value
	n = 15	n = 15	n = 30	
	n (%)	n (%)	n (%)	
*Gender*				0.999 ^a^
Female	12 (80.0)	12 (80.0)	24 (80.0)	
Male	3 (20.0)	3 (20.0)	6 (20.0)	
*Age (years)*				0.618 ^c^
Mean (SD)	79.5 (11.3)	80.9 (9.7)	80.2 (10.4)	
Median (min-max)	77 (65–98)	82 (65–98)	79.5 (65–98)	
*Fracture classification*				0.705 ^b^
Transtrochanteric (extracapsular) fractures	9 (60.0)	10 (66.7)	19 (63.3)	
Femoral neck (intracapsular) fractures	6 (40.0)	5 (33.3)	11 (36.7)	

**Abbreviations:** SD: standard deviation; max: maximum value; min: minimum value. **Notes:** ^a^ Fisher exact test. ^b^ Pearson chi-square test. ^c^ Mann-Whitney test.

Pain evaluation using the PAINAD scale for each treatment group and period showed no significant difference between the groups during hospitalization (a period with no medication or block). In contrast, after drug administration or PENG block, there was a significant difference between groups ( *p* < 0.05) for all periods evaluated (45 min, 12, 24, and 36 h) both at rest and during movement ( *p* ≤ 0.05) ( Table 3 ).

**Table 3:** The PAINAD scale of study participants per treatment group and evaluation time during rest or movement

Characteristic		Control	Intervention	***p*** ** -value ^a^**
		n = 15	n = 15	
		n (%)	n (%)	
*Rest*				
* Hospitalization*	Mean (SD)	2.0 (1.3)	1.7 (1.6)	0.464
	Median (Q _1_ -Q _3_ )	2 (1–3)	1 (1–3)	
* 45 minutes*	Mean (SD)	1.7 (1.2)	0.5 (0.8)	0.001
	Median (Q _1_ -Q _3_ )	1 (1–2)	0 (0–1)	
* 12 hours*	Mean (SD)	1.9 (1.4)	0.3 (0.8)	<0.001
	Median (Q _1_ -Q _3_ )	2 (1–3)	0 (0–0)	
* 24 hours*	Mean (SD)	2.0 (1.5)	0.3 (0.8)	0.001
	Median (Q _1_ -Q _3_ )	2 (1–3)	0 (0–0)	
* 36 hours*	Mean (SD)	2.0 (1.5)	0.9 (1.2)	0.046
	Median (Q _1_ -Q _3_ )	2 (1–3)	1 (0–1)	
*Movement*				
* Hospitalization*	Mean (SD)	6.2 (1.5)	7.3 (1.5)	0.051
	Median (Q _1_ -Q _3_ )	6 (5–8)	8 (6–8)	
* 45 minutes*	Mean (SD)	6.1 (1.2)	3.6 (2.1)	0.001
	Median (Q _1_ -Q _3_ )	6 (5–7)	3 (2–5)	
* 12 hours*	Mean (SD)	6.5 (1.2)	3.1 (2.0)	<0.001
	Median (Q _1_ -Q _3_ )	6 (5–7)	3 (2–4)	
* 24 hours*	Mean (SD)	6.5 (1.2)	3.8 (1.6)	<0.001
	Median (Q _1_ -Q _3_ )	6 (6–7)	4 (2–5)	
* 36 hours*	Mean (SD)	6.3 (1.3)	4.3 (1.1)	0.001
	Median (Q _1_ -Q _3_ )	6 (5–7)	4 (4–5)	

**Abbreviations:** PAINAD, pain assessment in advanced dementia; SD, standard deviation; Q1, first quartile; Q3, third quartile. **Note:** ^a^ Mann-Whitney test.

Comparison of PAINAD variation during hospitalization with the postmedication or block times showed a statistical difference between groups at rest ( *p* ≤ 0.05), except for the 36-hour timepoint. The movement assessment presented a statistical difference between groups at all times compared to hospitalization ( *p* < 0.001) ( Table 4 ).

**Table 4:** The PAINAD scale comparison between hospitalization and times per treatment group and evaluation time during rest or movement

Comparison				Control	Intervention	*p* -value ^a^
*Rest*	Hospitalization	45 minutes	Mean (SD)	-0.27 (0.80)	-1.20 (1.32)	0.044
			Median (Q _1_ -Q _3_ )	0 (-1; 0)	-1 (-2; 0)	
		12 hours	Mean (SD)	-0.07 (1.16)	-1.47 (1.36)	0.008
			Median (Q _1_ -Q _3_ )	0 (-1; 1)	-1 (-2; 0)	
		24 hours	Mean (SD)	0 (1.00)	-1.40 (1.35)	0.004
			Median (Q _1_ -Q _3_ )	0 (-1; 1)	-1 (-2; 0)	
		36 hours	Mean (SD)	0 (1.60)	-0.80 (1.37)	0.117
			Median (Q _1_ -Q _3_ )	0 (-1; 1)	0 (0; 1)	
***Movement***	**Hospitalization**	45 minutes	Mean (SD)	-0.13 (0.74)	-3.73 (1.79)	<0.001
			Median (Q _1_ -Q _3_ )	0 (0; 0)	-4 (-4; -2)	
		12 hours	Mean (SD)	0.27 (0.96)	-4.27 (2.19)	<0.001
			Median (Q _1_ -Q _3_ )	0 (0; 1)	-4 (-5; -3)	
		24 hours	Mean (SD)	0.33 (1.23)	-3.53 (1.85)	<0.001
			Median (Q _1_ -Q _3_ )	1 (0; 1)	-4 (-4; -2)	
		36 hours	Mean (SD)	0.07 (1.53)	-3.00 (1.41)	<0.001
			Median (Q _1_ -Q _3_ )	0 (-1; 1)	-3 (-4; -2)	

**Abbreviations:** PAINAD, pain assessment in advanced dementia; SD, standard deviation; Q1, first quartile; Q3, third quartile. **Note:** ^a^ Mann-Whitney test.

We noted a reduction in pain by 50% or more between groups and assessment times. The control group evaluated at rest demonstrated the desired pain reduction (≥ 50%) in 13.3, 20, 13.3, and 20% of patients, respectively, at each evaluation time (45 min, 12, 24, and 36 h). The intervention group presented, in these respective periods, 46.7, 66.7, 60, and 33.3% of patients with desired pain improvement (≥ 50%), with a significant difference for the 12 and 24-hour timepoints ( *p* < 0.05).

**Fig. 8:**
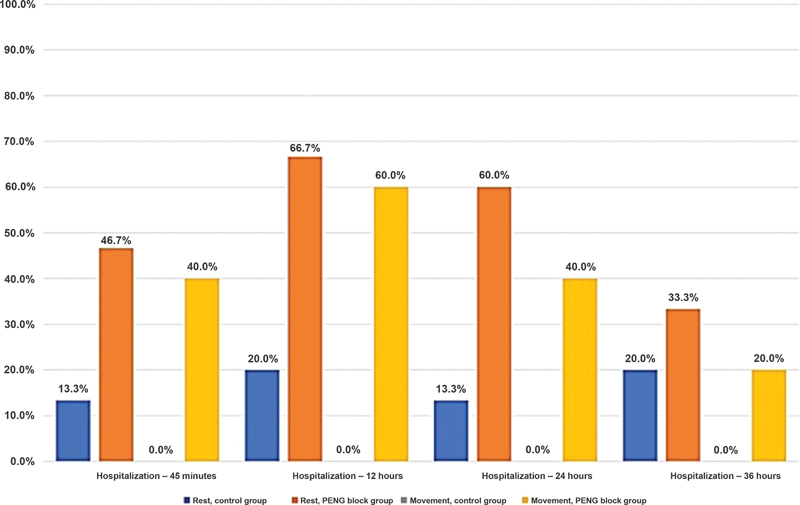
Graphical representation of the percentage of patients with pain improvement ≥50% at rest and movement per treatment group and evaluation period. **Abbreviation:** PENG, pericapsular nerve group.

During movement, no patient from the control group presented pain reduction ≥ 50%. In contrast, the intervention group demonstrated pain reduction in 40, 60, 40, and 20% of patients at 45 minutes, 12, 24, and 36 hours after the PENG block, respectively ( *p* ≤ 0.05, except for the 36-hour timepoint) ( Table 5 , Fig. 8 ).

**Table 5:** Pain reduction ≥ 50% and < 50%, per the PAINAD scale at rest or movement per treatment group and evaluation time

	Hospitalization	PAINAD	Control	Intervention	*p* -value ^a^
*Rest*	45 min	<50%	13 (86.7)	8 (53.3)	0.109
		≥50%	2 (13.3)	7 (46.7)	
	12 h	<50%	12 (80.0)	5 (33.3)	0.025
		≥50%	3 (20.0)	10 (66.7)	
	24 h	<50%	13 (86.7)	6 (40.0)	0.021
		≥50%	2 (13.3)	9 (60.0)	
	36 h	<50%	12 (80.0)	10 (66.7)	0.682
		≥50%	3 (20.0)	5 (33.3)	
*Movement*	45 min	<50%	15 (100)	9 (60.0)	0.017
		≥50%	0	6 (40.0)	
	12 h	<50%	15 (100)	6 (40.0)	0.001
		≥50%	0	9 (60.0)	
	24 h	<50%	15 (100)	9 (60.0)	0.017
		≥50%	0	6 (40.0)	
	36 h	<50%	15 (100)	12 (80.0)	0.224
		≥50%	0	3 (20.0)	

**Abbreviation:** NA, not available; PAINAD, pain assessment in advanced dementia. **Note:** ^a^ Fisher exact test.

**Fig. 9:**
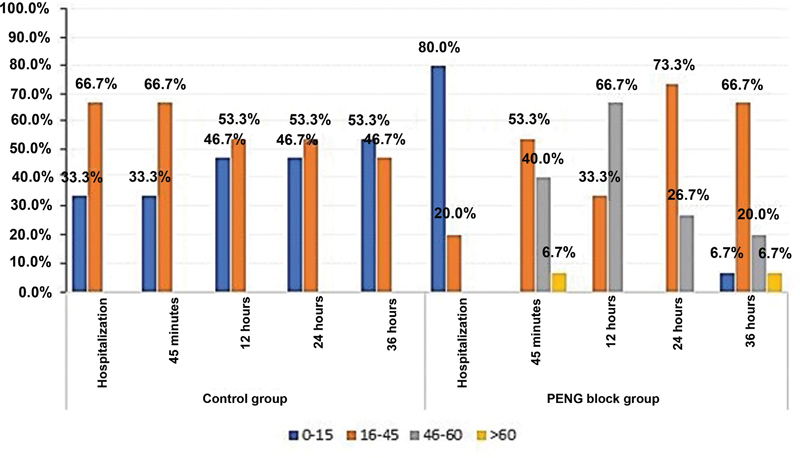
Graphical representation of the frequency of tolerable degree of hip flexion (0–15, 16–45, 46–60, and >60°) per treatment group and evaluation period. **Abbreviation:** PENG, pericapsular nerve group.

Tolerable hip flexion had a statistical difference between groups and assessment times ( *p* < 0.05) ( Table 6 , Fig. 9 ).

**Table 6:** Tolerable degree of hip flexion and strata range (0–15, 16–45, 46–60, and > 60°) per treatment group and evaluation time

Characteristic		Control	Intervention	***p*** **-value**
		n = 15	n = 15	
		n (%)	n (%)	
*Hospitalization*				
* Tolerable hip flexion measured in degrees*	Mean (SD) Median (Q _1_ -Q _3_ )	20.7 (8.8) 20 (15-20)	13.9 (10.5) 10 (8–15)	0.022 ^a^
* Flexion degree*	0–15	5 (33.3)	12 (80.0)	0.010 ^c^
	16–45	10 (66.7)	3 (20.0)	
*45 minutes*				
* Tolerable hip flexion measured in degrees*	Mean (SD)	20.3 (8.5)	47.0 (14.6)	<0.001 ^a^
	Median (Q _1_ -Q _3_ )	20 (15–25)	45 (40–60)	
* Flexion degree*	0–15	5 (33.3)	0	0.002 ^b^
	16–45	10 (66.7)	8 (53.3)	
	46–60	0	6 (40.0)	
	>60	0	1 (6.7)	
*12 hours*				
* Tolerable hip flexion measured in degrees*	Mean (SD)	19.0 (10.2)	49.7 (10.1)	<0.001 ^a^
	Median (Q _1_ -Q _3_ )	20 (10–30)	50 (45–60)	
* Flexion degree*	0–15	7 (46.7)	0	<0.001 ^b^
	16–45	8 (53.3)	5 (33.3)	
	46–60	0	10 (66.7)	
*24 hours*				
* Tolerable hip flexion measured in degrees*	Mean (SD)	18.7 (9.3)	42.7 (10.5)	<0.001 ^a^
	Median (Q _1_ -Q _3_ )	20 (10–25)	45 (40–50)	
* Flexion degree*	0–15	7 (46.7)	0	0.002 ^b^
	16–45	8 (53.3)	11 (73.3)	
	46–60	0	4 (26.7)	
*36 hours*				
* Tolerable hip flexion measured in degrees*	Mean (SD)	19.2 (8.2)	43.3 (11.3)	<0.001 ^a^
	Median (Q _1_ -Q _3_ )	15 (15–25)	45 (40–50)	
* Flexion degree*	0–15	8 (53.3)	1 (6.7)	0.011 ^b^
	16–45	7 (46.7)	10 (66.7)	
	46–60	0	3 (20.0)	
	>60	0	1 (6.7)	

**Abbreviations:** SD, standard deviation; NA, not available; Q1, first quartile; Q3, third quartile. **Notes:** ^a^ Mann-Whitney test. ^b^ Fisher exact test. ^c^ Pearson chi-square test.

**Fig. 10:**
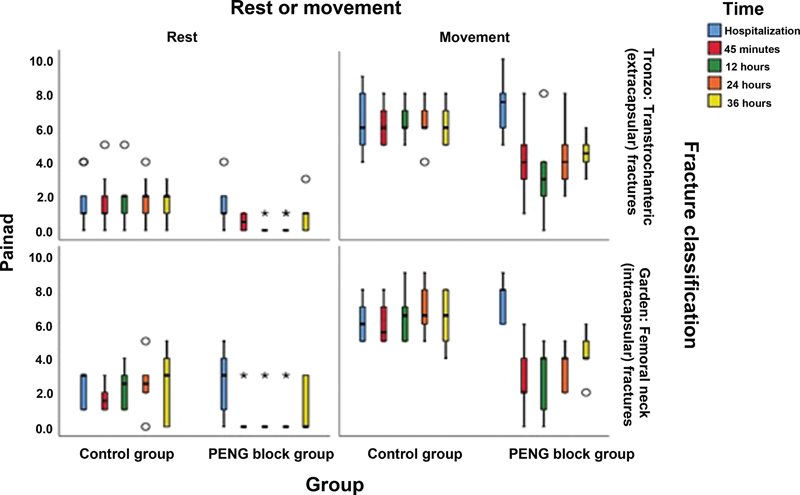
Graphical representation of the PAINAD scale per fracture type, treatment group, and evaluation period. **Abbreviations:** PAINAD, pain assessment in advanced dementia; PENG, pericapsular nerve group.

Pain and fracture type showed no difference between groups ( Fig. 10 ).

## Discussion

This study demonstrated that the preoperative PENG block performed in the emergency sector provided significant analgesia in elderly subjects with hip fractures both at rest and in movement, in addition to favoring a tolerable degree of flexion of the fractured hip.

Hip analgesic block techniques only had a moderate effect, not adequately covering the obturator nerve. 24 International guidelines question whether these blocks are relevant when compared to systemic analgesia. 25

Girón-Arango et al., 14 in 2018, demonstrated with their innovative technique that the PENG block for hip fractures provided a significant pain reduction. 14 Subsequently, clinical trials corroborated with similar outcomes. 26 27 This technique presented low complexity and risks, confirming it is safe and effective, and providing less motor blockade. 28 Even so, we must be attentive to the arguments regarding the need for more clinical trials to assure its safety. 29

Our study selected the PENG block considering its anatomical basis and development aiming analgesia in hip fractures with excellent preliminary outcomes. The PENG block is technically simple and can occur in the emergency sector. Reports of its preoperative performance in the emergency sector for elderly subjects remain scarce, and our study corroborates its practice.

Elderly subjects experience pain in a complex, multidimensional way requiring multidisciplinary management. 30 In a study on pain treatment, it is recommended to use a single measurement scale. We must consider what clinically significant reduction is ideal for treating pain and calculate it using percentage, not absolute reduction. Therefore, a pain reduction ranging from 30 to 33% is clinically significant. 31 32 33 34 35

Even using a more rigorous method, considering a clinically significant pain reduction of 50% or more, our study demonstrated that, at rest, over 12 hours, more than 65% of patients undergoing PENG block reached that goal, in contrast with 20% of subjects from the control group. During movement, in the same period, 60% of patients from the intervention group reported pain reduction by 50% or more, but none from the control group stated the same.

The study also aimed to improve the tolerable degree of hip flexion (≥45°), favoring greater mobility and comfort and, as a result, facilitating assistance with basic care, such as hygiene and nutrition. We noted an excellent, especially at 12-hour postblock, with more than 66% of patients from the intervention group demonstrating hip flexions of 46 to 60°, in contrast to no control patient.

## Conclusion

Elderly subjects with hip fractures undergoing the PENG block as preoperative additional analgesia experienced reduced pain and a better degree of tolerable hip flexion compared to those who received only standardized intravenous systemic analgesia. This method should be considered in the preoperative analgesia of elderly subjects with hip fractures awaiting definitive surgical treatment.
