# Supplementary material for: Pericapsular Hip Block Guided by Ultrasonography in Elderly People with Hip Fracture in the Emergency Sector: Clinical Trial
Source: Rev Bras Ortop (Sao Paulo). 2024 Apr 10;59(2):e284–96. doi: 10.1055/s-0044-1785494 (PMC11007597; doi:10.1055/s-0044-1785494)
Supplement: Related texts

# Research article: Bloqueio pericapsular do quadril guiado por ultrassonografia em idosos com fratura do quadril no setor de emergência: Ensaio clínico

**Keywords:** analgesia, anestesia por condução, bloqueio nervoso, dor, fraturas do quadril, ultrassonografia

## Abstract

**Objetivos** Este estudo avaliou a intensidade da dor em idosos acometidos por fratura do quadril internados no setor de emergência e submetidos ao *Pericapsular Nerve Group* (PENG) *block* no pré-operatório. Ademais, o grau de flexão tolerável do quadril foi avaliado.

**Métodos** Ensaio clínico, prospectivo, aleatorizado e controlado em grupos paralelos. O grupo controle consiste em idosos com fratura do quadril, submetidos à analgesia sistêmica endovenosa padronizada. O grupo intervenção consiste em idosos com fratura do quadril submetidos ao PENG *block* e analgesia sistêmica padronizada. Os grupos foram avaliados em repouso e durante o movimento pela escala de dor *Pain Assessment in Advance Dementia* (PAINAD). Aferiram-se intensidade da dor e redução álgica, assim como o grau de flexão tolerável do quadril fraturado. Todos os pacientes foram avaliados previamente à administração de medicação ou bloqueio e aos 45 minutos, 12, 24 e 36 horas pós-medicação ou bloqueio.

**Resultados** No pré-operatório e 24 horas após o PENG *block* , idosos com fratura do quadril apresentaram redução significativa da dor em repouso ou movimento em comparação com o controle ( *p* < 0,05), com 60% dos pacientes avaliados em repouso, demonstrando a redução álgica desejável de ≥ 50% e apenas 13,3% do grupo controle com redução álgica desejável. Durante o movimento, após o PENG *block* , 40% demonstraram redução álgica desejada e nenhum paciente do grupo controle apresentou a redução desejada. Verificou-se, também, no grupo intervenção a melhora significativa da flexão tolerável do quadril (p < 0,05).

**Conclusão** O PENG *block* no pré-operatório de idosos com fratura do quadril, internados no setor de emergência, proporcionou redução significativa da dor em comparação ao grupo controle.

## Introdução

As fraturas do fêmur proximal são frequentes na população idosa. 1 Sua prevalência aumenta frente à crescente longevidade populacional, 2 com incidência mundial estimada para 6,3 milhões de idosos acometidos no ano de 2050. 3

Trata-se de uma emergência ortopédica associada à significativa mortalidade e morbidade e requer tratamento cirúrgico bem como analgesia adequada. 3 4 Recomenda-se a cirurgia, preferencialmente nas primeiras 24 a 48 horas, por demonstrar alívio na dor e redução na incidência de complicações pós-operatórias e mortalidade. 5 6

Infelizmente, não é raro encontrarmos, em serviços públicos de emergência, idosos com fratura de quadril acamados à espera do tratamento cirúrgico definitivo dessas lesões e apresentando dor de forte intensidade. Neste período pré-operatório, a administração de opioides para alívio da dor consiste em terapêutica usual, mesmo associada a diversos efeitos colaterais, como náusea, vômito, constipação, hipotensão, sonolência e alteração mental. Menos comum, porém ainda mais preocupante, é que alguns pacientes podem desenvolver *delirium* , depressão respiratória e morte. 4 7 Por outro lado, o temor dessas complicações por parte da equipe médica e de enfermagem pode elevar o risco de oligoanalgesia. O uso de opioides em idosos deve ser balanceado tendo em vista seu potencial deletério e a ineficiência quando administrados isoladamente por via parenteral. 8 O controle inadequado da dor ou uso exagerado de opioide estão diretamente relacionados ao estado confusional agudo 8 que, por sua vez, quando associado à fratura do quadril, pode praticamente dobrar a taxa de mortalidade desta população no período de um ano. 9

Portanto, o tratamento da dor, além de ser questão humanitária, impacta a boa evolução desses pacientes. A dor está associada ao aumento da resposta ao estresse neuro-hormonal, isquemia miocárdica e atraso na recuperação e mobilização desses pacientes. 7 A revisão da literatura enfatiza que a analgesia na população idosa deve focar em minimizar os fatores de risco para o *delirium* , incluindo a dor, constipação e efeitos colaterais deliriogênicos. 10

Dentro deste contexto, a anestesia regional para o manejo da dor aguda está cada vez mais presente nos setores ou departamento de emergência, 8 demonstrando melhor eficácia em comparação à analgesia tradicional disponibilizada aos pacientes com fratura do quadril. 4 Os bloqueios regionais na região do quadril, efetuados no setor de emergência, demonstraram benefícios na redução da dor e do uso de opioides, podendo ser recomendados segundo revisão sistemática. 11 Não obstante, a associação com a ultrassonografia (USG) contribui para a eficiência desta técnica. 4 12

Para obtenção de analgesia eficaz do quadril, deve-se compreender o aspecto anatômico da cápsula articular. A região anterior da capsula é inervada por ramos do nervo femoral (NF), obturatório (NO) e obturatório acessório (NOA), proporcionando a maior contribuição da inervação sensitiva da articulação do quadril, sugerindo, portanto, que estes devam ser os principais ramos a serem bloqueados. 13 14 15 ( Fig. 1 e 2 )

**Fig. 1:** Inervação da região anterior da cápsula articular do quadril. **Abreviaturas:** NF, nervo femoral; NO, nervo obturatório; NOA, nervo obturatório acessório; NOBA, nervo obturatório, braço anterior; NOBP, nervo obturatório, braço posterior. 15

**Fig. 2:** Inervação da região posterior da cápsula articular do quadril. **Abreviaturas:** NGI, nervo glúteo inferior; NGS, nervo glúteo superior; NI, nervo isquiático; NQF, nervo quadrado femoral. 15

Girón-Arango et al., 14 baseando-se nesta informação anatômica e considerando que os principais ramos do NF e NOA são constantemente encontrados entre a espinha ilíaca anteroinferior e a eminência iliopúbica, descreveram uma técnica de bloqueio desses ramos capsulares do quadril guiado por USG, conhecida como *Pericapsular Nerve Group* (PENG) *block.* 14 15 16 ( Fig. 3 e 4 )

**Fig. 3:** Localização anatômica do PENG *block* em representação tridimensional de hemipelve e quadril direito. **Abreviaturas:** AF, artéria femoral; EIAI, espinha ilíaca anteroinferior; EIP, eminência iliopúbica; I, músculo Ilíaco; LI, ligamento inguinal; NF, nervo femoral; NO, nervo obturatório; NOA, Nervo obturatório acessório; P, Músculo psoas maior; VF, Veia femoral;  Ponto de infusão do anestésico. 15

**Fig. 4:** Localização anatômica do PENG *block* em representação tridimensional de quadril direito (Corte transversal). **Abreviaturas:** AF, artéria femoral; I, músculo ilíaco; LI, ligamento inguinal; NF, nervo femoral; NO, nervo obturatório; P, músculo psoas maior; VF, veia femoral;  Ponto de infusão do anestésico. 15

Evidências de relato do PENG *block* no pré-operatório de idosos acometidos por fratura do quadril, performado no setor de emergência de serviço público, são escassas. Neste cenário, o presente estudo considera que este bloqueio poderia auxiliar na redução da dor e mobilização destes pacientes em comparação à analgesia sistêmica padronizada.

O objetivo primário deste estudo consiste em avaliar a intensidade da dor nos idosos acometidos por fratura do quadril internados em setor de emergência e submetidos ao PENG *block* no período pré-operatório e, secundariamente, avaliar o grau de flexão tolerável do quadril acometido.

## Material e Métodos

Este ensaio clínico prospectivo, randomizado e controlado, dividido em dois braços paralelos, foi inscrito na Plataforma Brasil e aprovado pelo Comitê de Ética em Pesquisa (Protocolo: 38115120.4.0000.5479). O Termo de Consentimento Livre e Esclarecido foi assinado por todos os pacientes ou responsáveis legais. O trabalho foi registrado sob o número RBR-2zdn8pb no Registro Brasileiro de Ensaios Clínicos (REBEC).

Utilizando-se amostragem probabilística estratificada, os pacientes foram selecionados de forma sequencial entre idosos internados no setor de emergência de hospital público com diagnóstico radiográfico de fratura do quadril. Considerou-se um erro tipo I de 0,05 sendo *p1* = 0,05 (proporção de indivíduos do grupo controle, com redução da dor ao movimento em 50% ou mais) e *p2* = 0,5 (proporção de indivíduos do grupo intervenção com redução da dor em movimento de 50% ou mais) e um poder de teste de 0,80. O número total para pesquisa foi de 30 pacientes. 17 No entanto, considerando a taxa de desistência de 5%, o número final ideal para o estudo correspondeu a 32 pacientes ( Tabela 1 ).

**Tabela 1:** Tamanho de amostra para *p1* = 0,05 e *p2* = 0,50

Erro tipo I (α)	Poder estatístico (1-β)	* p _1 _* = 0,05 e * p _2_*_ _ = 0,50			
		n	n por grupo	+5%	
				n	n por grupo
	0,80	30	15	32	16

Ao todo, 59 pacientes com fratura do quadril foram abordados para eventual inclusão no período de março de 2020 a fevereiro de 2022.

## Critérios de Inclusão

Foram incluídos pacientes com idade maior ou igual a 65 anos, independente de gênero ou nível de cognição, diagnosticados radiograficamente com fratura aguda de colo femoral do tipo III ou IV baseando-se na classificação de Garden, 18 ou aqueles com fratura transtrocanteriana do tipo III a V segundo a classificação de Tronzo. 19 Também foram incluídos pacientes classificados pela *American Society of Anesthesiology* (ASA) como ASA II e III. 20

## Critérios de Exclusão

Foram excluídos pacientes com fraturas crônicas, patológicas, associação com outras fraturas, limitação prévia da flexão do quadril, antecedentes de alergia ou reações relatadas ao anestésico utilizado no bloqueio, ou com lesões cutâneas próximas ao sítio da punção. Excluíram-se, também, pacientes com insuficiência renal ou hepática avançada, aqueles em uso de anticoagulantes (dose terapêutica) ou distúrbio da coagulação prévios à fratura, além dos pacientes desacompanhados dos responsáveis legais.

Foram 27 pacientes excluídos e 32 randomizados em quatro blocos por sequência numérica aleatória gerada por computador 21 e depositada em envelope lacrado. O envelope foi aberto e o sorteio realizado por um pesquisador não vinculado ao estudo, no momento da internação do participante. Após o sorteio, pacientes e pesquisadores sabiam o grupo de alocação, tratando-se de estudo aberto. O PENG *block* foi realizado apenas pelo pesquisador responsável, enquanto a coleta dos dados pelo pesquisador e médico residente com treinamento prévio.

Ao todo, 16 pacientes foram alocados em grupo controle e 16 em grupo intervenção. Ao final, 15 pacientes de cada grupo foram avaliados, totalizando 30 participantes ( Fig. 5 ).

**Fig. 5:** Fluxograma CONSORT 2010.

## Variáveis Estudadas

- Dados sociodemográficos e tipos de fratura.
- Pontuação da escala *Pain Assessment in Advance Dementia* (PAINAD) em repouso e movimento.
- Grau de flexão tolerável do quadril fraturado.

Todos os pacientes foram submetidos ao mesmo formulário que incluía:

a) Dados sociodemográficos e clínicos: como idade, gênero, sinais vitais, doenças pré-existentes, medicações de uso contínuo, tratamento prévio, antecedentes pessoais, hábitos e vícios, alergia medicamentosa, tipo de fratura, sinais de toxicidade sistêmica, efeitos adversos, ou complicações.
b) Avaliação da dor foi feita com base na escala PAINAD, 22 traduzida e validada, a qual consiste em escala observacional composta por cinco itens: respiração, vocalização negativa, expressões sociais, linguagem corporal e consolabilidade. Cada item é classificado de 0 a 2 e, somados, variam entre 0 e 10 pontos. 23
c) Flexão tolerável do quadril: aferida por goniômetro no quadril fraturado durante a flexão passiva e assistida, com rotação neutra e interrompida por fácies de dor, resistência ao movimento ou aquele que ocorrer primeiro. A medida mensurada foi inserida em estratos de flexões de 0 a 15, 16 a 45, 46 a 60 e > 60° ( Fig. 6 ).

**Fig. 6:** Aferição da flexão tolerável do quadril através do goniômetro.

## Grupo Controle

Os participantes foram avaliados em repouso e movimento previamente à administração de qualquer medicação através do formulário da pesquisa com o registro da intensidade da dor e flexão tolerável do quadril.

Posteriormente, receberam analgesia endovenosa sistêmica (tramadol 100 mg e dipirona sódica 1g de 8/8h e 6/6h, respectivamente), sendo reavaliado em repouso e movimento após 45 minutos, 12, 24 e 36 horas.

## Grupo Intervenção

Previamente ao bloqueio, este grupo foi avaliado igualmente ao de controle. Posteriormente, foi encaminhado à sala de procedimento no setor de emergência para realização do *PENG block* guiado por USG. Após assepsia e antissepsia do quadril fraturado, o pesquisador paramentado de máscara e luva estéril realizou a colocação de campo e proteção estéril para o transdutor curvilíneo de baixa frequência do ultrassom modelo HS30, código de identificação US591 (Samsung Ltd., Suwon, Coréia do Sul) e manutenção regular. Com o paciente em posição supina, sob visualização ultrassonográfica, identificou-se a espinha ilíaca anterosuperior (EIAS), espinha ilíaca anteroinferior (EIAI), eminência iliopectínea (EIP), artéria e veia femorais e tendão do psoas. Referências anatômicas confirmadas, um botão anestésico foi realizado prosseguindo para introdução de agulha de 100mm e 22G de lateral para medial passando justa lateral e inferiormente aos psoas alcançando EIP. Após aspiração negativa excluindo-se a introdução intravascular, 25ml de solução anestésica de levobupivacaína a 0,25% foram ministrados ( Fig. 7 ).

**Fig. 7:** Ultrassonografia e preparo do paciente para o PENG *block.* ( **A** ) Aparelho de ultrassom utilizado no estudo. ( **B** ) Transdutor curvilíneo utilizado no estudo. ( **C** ) Região da hemipelve do paciente com campos estéreis para receber o bloqueio. ( **D** ). Imagem do ultrassom durante o procedimento. ( **E** ). Material utilizado para proceder o PENG *block* . **Abreviaturas:** A, ponto de aplicação do anestésico local; AF, artéria femoral; EIAI, espinha ilíaca anteroinferior; EIAS, espinha ilíaca anterossuperior; EIP, eminência iliopúbica; MP, músculo psoas maior.

Concluído o PENG *block* , passados 45 minutos, 12, 24 e 36 horas, aplicou-se novamente o questionário da pesquisa em repouso e durante o movimento, registrando a intensidade da dor e grau de flexão tolerável do quadril para cada período avaliado. Este grupo, adicionalmente, recebeu a mesma analgesia sistêmica do controle.

## Desfecho Primário e Secundário

Redução na intensidade da dor ≥ 50% utilizando-se a escala PAINAD no pré-operatório de idosos com fratura do quadril submetidos ao PENG *block* e melhora no grau de flexão tolerável do quadril ≥ 45°, avaliado por goniômetro.

## Análise Estatística

As características dos pacientes foram apresentadas pelas frequências absolutas e relativas (para as variáveis qualitativas) e da média, mediana, desvio padrão (DP), valores mínimo e máximo, assim como o primeiro e terceiro quartis.

A comparação entre grupos independentes foi avaliada pelo teste de Mann-Whitney (variável quantitativa). A associação entre variáveis qualitativas foi avaliada pelo teste qui-quadrado de Pearson ou pelo teste exato de Fisher.

Na avaliação das frequências, antes e depois em relação ao grau de flexão, utilizou-se o teste de McNemar.

O nível de significância adotado foi de 5% para todos os testes de hipóteses e as análises foram realizadas no software estatístico Statistical Package Social Sciences (SPSS, IBM Corp. Armonk, NY, USA) para Windows, v.25. Os resultados foram apresentados de acordo com os objetivos do estudo:

- Comparação da escala de dor PAINAD entre grupos e tempos.
- Comparação da variação PAINAD entre os tempos por grupo.
- Comparação da redução da dor ≥ 50% entre os grupos e tempo.
- Avaliação do grau de flexão entre os grupos e tempos.
- Comparação da variação PAINAD e os tipos de fratura.
- Descrição das características sociodemográficas e clínicas dos participantes do estudo segundo o tratamento assinado.
- Representação gráfica dos resultados.

## Resultados

As características sociodemográficas e clínicas não apresentaram diferença estatística significativa ( Tabela 2 ).

**Tabela 2:** Características dos pacientes participantes do estudo segundo o grupo de tratamento

Característica	Controle	Intervenção	Total	Valor de p
	n = 15	n = 15	n = 30	
	n (%)	n (%)	n (%)	
*Sexo*				0,999 ^a^
Feminino	12 (80,0)	12 (80,0)	24 (80,0)	
Masculino	3 (20,0)	3 (20,0)	6 (20,0)	
*Idade (anos)*				0,618 ^c^
Média (DP)	79,5 (11,3)	80,9 (9,7)	80,2 (10,4)	
Mediana (mín-máx)	77 (65–98)	82 (65–98)	79,5 (65–98)	
*Classificação das fraturas*				0,705 ^b^
Fraturas transtrocanterianas (extracapsulares)	9 (60,0)	10 (66,7)	19 (63,3)	
Fraturas de colo do fêmur (intracapsulares)	6 (40,0)	5 (33,3)	11 (36,7)	

**Abreviaturas:** DP: desvio padrão; máx: valor máximo; mín: valor mínimo. **Notas:** ^a^ Teste exato de Fisher. ^b^ Teste qui-quadrado de Pearson. ^c^ Teste de Mann-Whitney.

Ao avaliar a dor na escala PAINAD para cada grupo de tratamento e período considerado, verificou-se que na internação (período no qual não há medicação ou bloqueio) não houve diferença significativa entre os grupos. Em contraste, após administração medicamentosa ou PENG *block,* verificou-se diferença significativa entre os grupos ( *p* < 0,05) para todos os períodos avaliados (45min, 12, 24 e 36h), tanto em repouso quanto em movimento ( *p* ≤ 0,05) ( Tabela 3 ).

**Tabela 3:** Escala PAINAD dos participantes do estudo segundo grupo de tratamento, tempo avaliado em repouso e movimento

Característica		Controle	Intervenção	Valor de *p* ^a^
		n = 15	n = 15	
		n (%)	n (%)	
*Repouso*				
* Internação*	Média (DP)	2,0 (1,3)	1,7 (1,6)	0,464
	Mediana (Q _1_ -Q _3_ )	2 (1–3)	1 (1–3)	
* 45 minutos*	Média (DP)	1,7 (1,2)	0,5 (0,8)	0,001
	Mediana (Q _1_ -Q _3_ )	1 (1–2)	0 (0–1)	
* 12 horas*	Média (DP)	1,9 (1,4)	0,3 (0,8)	<0,001
	Mediana (Q _1_ -Q _3_ )	2 (1–3)	0 (0–0)	
* 24 horas*	Média (DP)	2,0 (1,5)	0,3 (0,8)	0,001
	Mediana (Q _1_ -Q _3_ )	2 (1–3)	0 (0–0)	
* 36 horas*	Média (DP)	2,0 (1,5)	0,9 (1,2)	0,046
	Mediana (Q _1_ -Q _3_ )	2 (1–3)	1 (0–1)	
*Movimento*				
* Internação*	Média (DP)	6,2 (1,5)	7,3 (1,5)	0,051
	Mediana (Q _1_ -Q _3_ )	6 (5–8)	8 (6–8)	
* 45 minutos*	Média (DP)	6,1 (1,2)	3,6 (2,1)	0,001
	Mediana (Q _1_ -Q _3_ )	6 (5–7)	3 (2–5)	
* 12 horas*	Média (DP)	6,5 (1,2)	3,1 (2,0)	<0,001
	Mediana (Q _1_ -Q _3_ )	6 (5–7)	3 (2–4)	
* 24 horas*	Média (DP)	6,5 (1,2)	3,8 (1,6)	<0,001
	Mediana (Q _1_ -Q _3_ )	6 (6–7)	4 (2–5)	
* 36 horas*	Média (DP)	6,3 (1,3)	4,3 (1,1)	0,001
	Mediana (Q _1_ -Q _3_ )	6 (5–7)	4 (4–5)	

**Abreviaturas:** DP, desvio padrão; PAINAD, *Pain Assessment in Advance Dementia;* Q _1_ , primeiro quartil; Q _3_ , terceiro quartil. **Notas:** ^a^ Teste de Mann-Whitney.

Ao comparar a variação PAINAD, no momento da internação, com os tempos pós-medicação ou bloqueio, verificou-se, em repouso, diferença estatística entre os grupos ( *p* ≤ 0,05), exceto no comparativo de internação com 36 horas. Na avaliação em movimento, houve diferença estatística entre os grupos, para todos os tempos comparados à internação ( *p* < 0,001) ( Tabela 4 ).

**Tabela 4:** Escala PAINAD - comparativo entre internação e tempos, segundo grupo de tratamento avaliado em repouso e movimento

Comparação				Controle	Intervenção	Valor de *p* ^a^
*Repouso*	Internação	45 minutos	Média (DP)	-0,27 (0,80)	-1,20 (1,32)	0,044
			** Mediana (Q _1_ -Q _3_ ) **	**0 (-1; 0)**	**-1 (-2; 0)**	
		**12 horas**	**Média (DP)**	**-0,07 (1,16)**	**-1,47 (1,36)**	**0,008**
			Mediana (Q _1_ -Q _3_ )	0 (-1; 1)	-1 (-2; 0)	
		24 horas	Média (DP)	0 (1,00)	-1,40 (1,35)	0,004
			Mediana (Q _1_ -Q _3_ )	0 (-1; 1)	-1 (-2; 0)	
		36 horas	Média (DP)	0 (1,60)	-0,80 (1,37)	0,117
			Mediana (Q _1_ -Q _3_ )	0 (-1; 1)	0 (0; 1)	
*Movimento*	Internação	45 minutos	Média (DP)	-0,13 (0,74)	-3,73 (1,79)	<0,001
			Mediana (Q _1_ -Q _3_ )	0 (0; 0)	-4 (-4; -2)	
		12 horas	Média (DP)	0,27 (0,96)	-4,27 (2,19)	<0,001
			Mediana (Q _1_ -Q _3_ )	0 (0; 1)	-4 (-5; -3)	
		24 horas	Média (DP)	0,33 (1,23)	-3,53 (1,85)	<0,001
			Mediana (Q _1_ -Q _3_ )	1 (0; 1)	-4 (-4; -2)	
		36 horas	Média (DP)	0,07 (1,53)	-3,00 (1,41)	<0,001
			Mediana (Q _1_ -Q _3_ )	0 (-1; 1)	-3 (-4; -2)	

**Abreviaturas:** DP, desvio padrão; PAINAD, *Pain Assessment in Advance Dementia;* Q _1_ , primeiro quartil; Q _3_ , terceiro quartil. **Notas:** ^a^ Teste de Mann-Whitney.

Uma redução do quadro álgico em 50% ou mais foi pesquisada entre os grupos e os tempos de avaliação. O grupo controle avaliado em repouso demonstrou redução desejada da dor (≥ 50%) em 13,3, 20, 13,3 e 20% dos pacientes, respectivamente ao tempo de avaliação (45 min, 12, 24 e 36h). O grupo intervenção apresentou no respectivo período 46,7, 66,7, 60 e 33,3% dos pacientes com melhora da dor desejada (≥50%), com uma diferença significativa para o período de 12 e 24 horas ( *p* < 0,05).

Durante movimento, nenhum paciente do grupo controle obteve redução na dor ≥ 50%. O grupo intervenção, por sua vez, demonstrou redução do quadro álgico em 40, 60, 40 e 20% dos pacientes, respectivamente, com o período 45 minutos, 12, 24 e 36 horas ( *p* ≤ 0,05, exceto em 36 horas) ( Tabela 5 , Fig. 8 ).

**Fig. 8:** Representação gráfica: porcentagem dos pacientes com melhora na dor ≥ 50% em repouso e movimento segundo grupo de tratamento e período de avaliação.

**Tabela 5:** Redução álgica ≥ 50% e <50% (variação PAINAD) em repouso e movimento pelo tempo de avaliação e grupo de tratamento

	Internação	PAINAD	Controle	Intervenção	Valor de *p* ^a^
*Repouso*	45 min	<50%	13 (86,7)	8 (53,3)	0,109
		≥50%	2 (13,3)	7 (46,7)	
	12 h	<50%	12 (80,0)	5 (33,3)	0,025
		≥50%	3 (20,0)	10 (66,7)	
	24 h	<50%	13 (86,7)	6 (40,0)	0,021
		≥50%	2 (13,3)	9 (60,0)	
	36 h	<50%	12 (80,0)	10 (66,7)	0,682
		≥50%	3 (20,0)	5 (33,3)	
*Movimento*	45 min	<50%	15 (100)	9 (60,0)	0,017
		≥50%	0	6 (40,0)	
	12 h	<50%	15 (100)	6 (40,0)	0,001
		≥50%	0	9 (60,0)	
	24 h	<50%	15 (100)	9 (60,0)	0,017
		≥50%	0	6 (40,0)	
	36 h	<50%	15 (100)	12 (80,0)	0,224
		≥50%	0	3 (20,0)	

**Abreviaturas:** NA, não avaliável; PAINAD, *Pain Assessment in Advance Dementia* . **Nota:** ^a^ Teste exato de Fisher.

Em relação à flexão tolerável do quadril, observou-se diferença estatística entre os grupos e seus devidos tempos ( *p* < 0,05) ( Tabela 6 , Fig. 9 ).

**Fig. 9:** Representação gráfica da frequência do grau de flexão tolerável do quadril (0–15, 16–45, 46–60 e >60°) em relação ao grupo e período de tratamento.

**Tabela 6:** Grau de flexão tolerável do quadril e variação por estratos de (0–15, 16–45, 46–60 e >60°) segundo grupo e tempo de tratamento

Característica		Controle	Intervenção	Valor de *p*
		n = 15	n = 15	
		n (%)	n (%)	
*Internação*				
* Flexão tolerável do quadril aferida em graus*	Média (DP) Mediana (Q _1_ -Q _3_ )	20,7 (8,8) 20 (15–20)	13,9 (10,5) 10 (8–15)	0,022 ^a^
* Grau de flexão*	0–15	5 (33,3)	12 (80,0)	0,010 ^c^
	16–45	10 (66,7)	3 (20,0)	
*45 minutos*				
* Flexão tolerável do quadril aferida em graus*	Média (DP)	20,3 (8,5)	47,0 (14,6)	<0,001 ^a^
	Mediana (Q _1_ -Q _3_ )	20 (15–25)	45 (40–60)	
* Grau de flexão*	0–15	5 (33,3)	0	0,002 ^b^
	16–45	10 (66,7)	8 (53,3)	
	46–60	0	6 (40,0)	
	>60	0	1 (6,7)	
*12 horas*				
* Flexão tolerável do quadril aferida em graus*	Média (DP)	19,0 (10,2)	49,7 (10,1)	<0,001 ^a^
	Mediana (Q _1_ -Q _3_ )	20 (10–30)	50 (45–60)	
* Grau de flexão*	0–15	7 (46,7)	0	<0,001 ^b^
	16–45	8 (53,3)	5 (33,3)	
	46–60	0	10 (66,7)	
*24 horas*				
* Flexão tolerável do quadril aferida em graus*	Média (DP)	18,7 (9,3)	42,7 (10,5)	<0,001 ^a^
	Mediana (Q _1_ -Q _3_ )	20 (10–25)	45 (40–50)	
* Grau de flexão*	0–15	7 (46,7)	0	0,002 ^b^
	16–45	8 (53,3)	11 (73,3)	
	46–60	0	4 (26,7)	
*36 horas*				
* Flexão tolerável do quadril aferida em graus*	Média (DP)	19,2 (8,2)	43,3 (11,3)	<0,001 ^a^
	Mediana (Q _1_ -Q _3_ )	15 (15–25)	45 (40–50)	
* Grau de flexão*	0–15	8 (53,3)	1 (6,7)	0,011 ^b^
	16–45	7 (46,7)	10 (66,7)	
	46–60	0	3 (20,0)	
	>60	0	1 (6,7)	

**Abreviaturas:** DP, desvio padrão; NA, não avaliável; Q _1_ , primeiro quartil; Q _3_ , terceiro quartil. **Notas** : ^a^ Teste de Mann-Whitney. ^b^ Teste exato de Fisher. ^c^ Teste qui-quadrado de Pearson.

Em relação à dor e tipo de fratura estudada, não houve diferença entre os grupos ( Fig. 10 ).

**Fig. 10:** Representação gráfica da variação da dor (PAINAD) entre os tipos de fratura, grupo de tratamento e tempo avaliado.

## Discussão

Neste estudo foi possível demonstrar que o PENG *block* realizado no setor de emergência proporcionou analgesia significativa em idosos com fratura do quadril no pré-operatório quando avaliados tanto em repouso quanto em movimento, além de favorecer o grau de flexão tolerável do quadril fraturado.

Técnicas de bloqueio analgésico do quadril evidenciaram efeito apenas moderado, não abrangendo adequadamente o nervo obturatório. 24 Diretrizes internacionais questionam se esses bloqueios são relevantes quando comparados à analgesia sistêmica. 25

Girón-Arango et al., 14 em 2018, demonstraram com sua técnica inovadora que o PENG *block* , quando utilizado em fraturas do quadril, proporcionou redução significativa da dor. 14 Posteriormente, ensaios clínicos corroboraram com resultados semelhantes. 26 27 Essa técnica apresentou baixa complexidade e risco envolvido, verificando segurança e eficácia e proporcionando menor bloqueio motor. 28 Mesmo assim, devemos estar atentos aos argumentos quanto à necessidade de mais ensaios clínicos para verificar a segurança do método. 29

Nosso estudo selecionou o PENG *block* considerando seu embasamento anatômico e seu desenvolvido objetivando analgesia em fraturas do quadril com excelentes resultados preliminares, sendo um método tecnicamente simples e passível de aplicação no setor de emergência. Os relatos de sua utilização no setor de emergência para idosos com fratura do quadril no pré-operatório ainda são escassos e nosso estudo corrobora com esta prática.

Experiências de dor em idosos são complexas, multidimensionais e requerem gestão multidisciplinar. 30 Ao realizar um estudo para o tratamento da dor, recomenda-se a utilização de apenas uma escala de aferição. Deve-se ter em mente qual a redução clinicamente significativa é ideal para o tratamento da dor e calculá-la utilizando redução percentual e não absoluta. Desta forma, redução álgica de 30 a 33% é considerada como clinicamente significativa. 31 32 33 34 35

Mesmo utilizando método mais rigoroso, considerando redução da dor clinicamente significativa de 50% ou mais, foi possível demonstrar que, em repouso, no período de 12 horas, mais de 65% dos pacientes submetidos ao PENG *block* alcançaram o objetivo, em contraste com 20% dos pacientes do grupo controle. Em movimento, no mesmo período, 60% dos pacientes do grupo intervenção reportaram redução de dor igual ou maior que 50%, mas ninguém do grupo controle apresentou o mesmo.

O estudo também objetivou a melhora no grau de flexão tolerável do quadril (≥ 45°), favorecendo maior mobilidade e conforto e, consequentemente, facilitando a assistência aos cuidados básicos como higiene e alimentação. Observou-se excelente resultado, principalmente no período de 12 horas após o bloqueio, com mais de 66% dos pacientes do grupo intervenção demonstrando flexões do quadril de 46 a 60°, contrastando com nenhum paciente controle.

## Conclusão

Idosos com fratura do quadril que receberam o PENG *block* como analgesia adicional no pré-operatório experimentaram nível reduzido de dor e melhor grau de flexão tolerável do quadril em comparação àqueles que receberam somente analgesia sistêmica endovenosa, padronizada. Esse método deve ser considerado na analgesia pré-operatória de idosos com fratura do quadril que aguardam tratamento cirúrgico definitivo.
